# The American Plan of Hospital Service

**Published:** 1921-04-02

**Authors:** 


### The Hospital

The Workers' Newspaper of Administrative Medicine and Institutional Life, Administration, National Insurance and Health.

No. 1817, Vol. LXX. SATURDAY, APRIL 2, 1921. PRICE TWOPENCE

### THE AMERICAN PLAN OF HOSPITAL SERVICE.

We have received from Mr. Donelda R. Hamlin, the director of the Hospital Library and Service Bureau of the American conference on hospital service, an interesting outline of the material which the former body plans to collect. It is suggestive, because, like all draft schemes for further co-operation and the interchange of information, it invites criticism, and can indeed be perfected only when any defects of the draft have been remarked by hospital officers, omissions suggested, and new headings supplied. For this reason we print the skeleton on another page that our readers may set their minds to work upon its improvement. If they will do this, not only will they aid their American colleagues, but help their own institutions in Great Britain at the very moment when co-operation is the aspiration of the day. It has been for lack of such intelligent interest, perhaps, that the Hospital Library and Bureau of Information lodged years ago at 28 Southampton Street, Strand, has not been more patronised, though it remains to this day always open to any responsible person. Students of the American scheme must not be disappointed that only a fraction of the material to be collected by the American Hospital Library is at present available. Announcements will be made as the material increases, and by a system of instalment catalogues, or rather statements, the matter available for reference will be announced. For this reason now is the time to make suggestions; and we shall be glad to publish any criticisms of the completeness of the proposed collection.

Before discussing the draft plan, we may remind our readers that the American Hospital Library and Service Bureau has been organised by the national hospital, public health, nursing and social services, with the help of the Rockefeller Foundation. Its object is to serve gratuitously all actively interested in institutional medical matters; and the policy to be pursued has been sketched by Dr. Frank Billings, Dean of the Rush Medical College, Chicago, who is also chairman of the Library Committee. Last October Dr. Billings delivered the presidential address at the joint meeting of the American Hospitals Association and the American Conference on Hospital Service at Montreal, Canada. After congratulating the meeting on the establishment of the Conference, of which the second gathering was then taking place, Dr. Billings remarked that its value lay in a common ideal "to improve the medical care of the sick and injured, disregardful of the too-frequent causes of non-co-operation through differences engendered by sex, race, religion, politics," and institutional inability. Then he turned to the brief for the library, drafted by Dr. A. R. Warner, whose first minute ran:

> The proposed Library and Service Bureau will collect, classify for reference, and distribute types of data as outlined below. Pamphlets and data will also be collected and filed in such form as to be readily available to make up bundles to be sent out in answer to inquiries.

The complete minute will be found on page 6. Dr. Billings then laid down certain principles: That a hospital's chief obligation is the welfare of the patient; that a hospital should be the health centre of the community which it serves; and that it should become a school of instruction on all the subjects in which it functions. He remarked that the American College of Surgeons had established a minimum hospital standard, primarily concerned with the character of the medical staff; the organisation of the (presumably lay) staff; the method of filing clinical records; and the holding of a monthly meeting to discuss and review there with the aid of a minimum fixed degree of laboratory and special apparatus. This standard, he said, was being widely accepted, and meanwhile other bodies have secured important data and laid down an appropriate standard for nurses' training-schools, medical social service, the study of industrial diseases, the relation of hospitals to both parties involved in causes under the Workmen's Compensation Acts. Thus various medical, institutional, architectural, and educational bodies are severally trying to fix a minimum hospital standard, so far as they and the hospitals interact upon each other. Into all this activity the Hospital Library and Service Bureau enters as the central point of rest, so to speak, to which the general radiating energies may be referred. They all have something to contribute. It is their common depository, and by its means their several difficulties, conclusions, and experiences may be

W1
H069X

made common property. If the library is regularly fed by these it will repay this service by classifying all the information.

That is the scheme, and since, we suppose, there can be no criticism of its object or usefulness, suggestions are rather sought on methods and means. In this case we fancy British institutions have more to learn from their American colleagues than they from us. Here co-operation is only in its infancy, because the voluntary spirit is the fruit of individualism, and hitherto the defect of its quality has been to make each hospital self-sufficient and sometimes aloof. The pressure of financial difficulty, however, has encouraged cohesion, and perhaps the chief value to us of this American scheme is the nourishment which it offers to a similar desire among ourselves. For this reason we commend it to the careful study of our readers and in particular to the newly formed regional committees of the British Hospitals Association.

